# A study on knowledge, attitude, and practice towards premarital carrier screening among adults attending primary healthcare centers in a region in Oman

**DOI:** 10.1186/1471-2458-14-380

**Published:** 2014-04-17

**Authors:** Omar A Al-Farsi, Yahya M Al-Farsi, Ishita Gupta, Allal Ouhtit, Khalil S Al-Farsi, Samir Al-Adawi

**Affiliations:** 1 Department of Family Medicine and Public Health, College of Medicine and Health Sciences, Sultan Qaboos University, Muscat, Oman; 2 Department of Genetics, College of Medicine and Health Sciences, Sultan Qaboos University, Muscat, Oman; 3 Department of Hematology, College of Medicine and Health Sciences, Sultan Qaboos University, Muscat, Oman; 4 Department of Behavioral Medicine, College of Medicine and Health Sciences, Sultan Qaboos University, Muscat, Oman

**Keywords:** Premarital carrier screening, Hereditary diseases, Oman

## Abstract

**Background:**

Despite that hereditary diseases are widespread among the Arab population due to high rates of consanguineous marriages, research regarding community awareness towards premarital carrier screening in some countries such as Oman, is extremely scarce. This study aimed to investigate knowledge and attitude towards premarital carrier screening (PMCS) in Oman.

**Methods:**

A cross-sectional study was conducted using a self-administered questionnaire which was distributed to 400 Omani adults aged 20–35 who attended primary healthcare institutions at the South Batinah Governorate in Oman.

**Results:**

The majority of the participants (84.5%) believed that PMCS was necessary, and about half of them (49.5%) supported the view of making PMCS compulsory. On the contrary, approximately one third (30.5%) of the participants reported that they were not in favor of taking the blood screening test. Overall, unwillingness to perform pre-marital testing was associated with female gender, younger age, being single, less education, and increased income.

**Conclusion:**

Despite the relatively high level of knowledge, about one third of the participants were still reluctant to carry out premarital testing. Such attitude calls for immediate need for community-based campaigns to encourage the public to do premarital testing.

## Background

Available data suggest that congenital disorders are more common in Arab countries than in their counterparts elsewhere [1]. Prior to the onset of the recent affluence, Arab children born with handicapping conditions were likely to have perished while still in their tender age [2]. However, modern medicine has reversed such trend; there is still a concern on the quality of life of some of those children born with persistent and pervasive handicapping conditions if they survived into adulthood. Within such background, discussion on the intervening role of premarital screening or premarital carrier screening (PMCS) comes to the forefront. PMCS and the resultant genetic counseling are increasingly recognized as integral part of the much-herald preventive medicine [3] which more recently, have been used increasingly to inoculate prospective parents of conceiving children with preventive deformities, if any. The emergence of PMCS has conjured the view on how to proceed in implementing premarital screening at the national level that could be acceptable to the society, as many traditional societies are shrouded with the social prescription that are akin to fatalism. Therefore, studies are needed to gauge the ‘pulse’ of the society towards PMCS.

Oman represents a fertile ground for such undertaking. Firstly, because of recent affluence, the country has one of the highest fertility rate which has rendered the country with ‘youth bulge’ and expected ‘baby boom’ [4] and the likelihood of conceiving children born with handicapping conditions. Secondly, Oman has been reported to be among the countries with high risk of having children born with handicapping conditions due to high rates of consanguineous marriages [5]. There is ample of empirical studies suggesting that consanguineous marriages increase the risk of hereditary diseases and genetic disorders [6]. In Oman, the rate of consanguineous marriage among ‘first cousins’ has been reported to be 34%, and 58% among second cousins [7]. Within the trajectory of baby boom, the high rates of consanguineous marriages and the risk of conceiving children with handicapping conditions, the question still remains with the possibility of introducing mandatory PMCS. If such quest would deem imperative, then studies are needed to shed light on the knowledge, attitude and practice towards PMCS in Oman. Previous studies have been limited to the college population of Sultan Qaboos University to find out the knowledge and attitude towards premarital screening program [8]. Most of the participants (79%) were aware to do premarital screening, but about half of them (53%) prefer to make it compulsory. Therefore, community studies will be essential to know the population knowledge towards such important issue.

In Oman, the PMCS service is available in all health centers and referral hospitals. The services in the country cover screening for the most common hemoglobinopathies namely sickle cell disease (SCD) and thalassemia. Through media campaigns, all individuals, especially couples who intend to wed are encouraged to the PMCS. Nonetheless, the service is non-compulsory. During the PMCS, individuals receive health education about hereditary disorders and are also subjected to do the blood test. If found to be carriers, the couples are counseled about the consequences of getting affected children sequel. They are also informed about the available technology that would assist in the prevention of having affected children, such as in-vitro fertilization and pre-conceptual genetic diagnosis (PGD). As PGD still does not exist in the country, they are provided with information about existing PGD centers in the neighboring countries where the service can be availed. After marriage, if the couples wish to get more information about the test, they are encouraged to contact the health centers and they would be directed to referral hospitals for further follow up if adverse outcomes of future pregnancies are anticipated.

In other countries such as Saudi Arabia where there is an increased rate of consanguineous marriage, premarital medical testing has been made compulsory for prospective couples [9]. In this country, couples who plan to marry undergo blood test to investigate if they are carriers of any of the genetic blood disorders: thalassemia, sickle cell anemia and Gloucose-6-phosphate dehydrogenase deficiency (G6PD) [10]. Genetic Blood Disorder Survey was conducted to estimate the prevalence rate of the most common genetic blood disorders among Omani children under the age of five in 1995 [7]. The prevalence of sickle cell disease was 6%, 2% for β-thalassemia and 25% for G6PD [7]. This would suggest that the country is marked with potential minefield of having to care for such debilitating and refractory conditions. There is dearth of literature from Arab/Islamic countries examining knowledge, attitude, and practice towards PMCS such as blood disorders. The present study aims to quantify the knowledge, attitude, and the beliefs towards PMCS in the Governorate of South Batinah, one representative region of Oman. This region had an estimated population of 315,351 [11] and is adjacent to the satellite town of the capital, Muscat -- the most populous region of the country. It attracts all the rural population of Oman by virtue of its location as the nation’s capital. If PMS would be endorsed by Omanis, then its implication would be enormous. As it has been reported from other countries, [12], [13] the country could contemplate to institute framework to develop national PMCS which could lay groundwork mitigating the burden of caring for people with such impervious to treatment blood disorders.

## Methods

This is a cross-sectional descriptive study carried out in 16 primary health care centers in the South Batinah Governorate. The study was conducted during the period September to December 2011.

The sample size has been estimated using EPI Info version 6.0 computer program. With a type-1 error of 5% (alpha = 0.05) and 95% level of significance, it was estimated that 460 subjects will be required in order for the study to detect a 50% difference in odds ratio (OR) at a power level equals 90%. Therefore, the target has been set to reach 460 participants in order to achieve the objective of the study.

Selection of the participants was done by simple random sampling among Omanis, aged 20–35 who visited the health centers for various reasons during the study period. The age-range selection criterion has been set to reflect the expected marital age in Oman. Potential candidates among visitors to health centers who fulfilled the inclusion criteria were approached and invited by four staff nurses who were trained to be the research assistants. The purpose of the study, as well as the methodology was explained in details. All potential participants received an information sheet that covers all aspects of the study, and their inquiries were responded to. Candidates who agreed to participate in the study were asked to sign a consent form, and were provided with contacts of the investigators for any further inquiries.

A self-administered questionnaire was used to collect the data under the supervision of qualified nurses and health educators. A total of 460 persons were approached, and 428 (93.0%) consented to participate in the study. Nonetheless, 28 questionnaires were excluded from the study due to incomplete responses. A total of 400 subjects were therefore included in the analysis.

Based on available literature on Knowledge Attitude Perception (KAP) paradigm on PMCS, a questionnaire was developed with items that would not contravene Oman’s socio-cultural teaching. Firstly, an English language version of the questionnaire was developed in order to elicit KAP towards PMCS. The questionnaire included both close- and open-ended questions. Close-ended questions, in a checklist format, were designed to investigate people's knowledge towards PMCS, while open-ended questions were designed to explore people's opinion towards the reasons for their answer selection. The questionnaire was composed of three main parts: socio-demographic data, participants' knowledge, and reasons that prevented them from taking the test in cases where they did not take it. The first part: socio-demographic data was concerned about age, gender, marital status, and educational level. The second part was concerned about the attitude towards PMCS. For example, we asked the participants if they think that the premarital carrier screening was important for unmarried individuals. The third part consisted of open-ended questions which helped investigate reasons that prevented them from taking the test. For example, the participants were asked about their beliefs if the PMCS can limit the spread of hereditary diseases. In addition, the questionnaire addressed the options available after PMCS, namely the options of choosing not to get married, getting married but not having a child with the screened partner, and PGD.

The questionnaire was piloted on 30 randomly-selected participants to assess its clarity, as well as to modify and update certain linguistic and succinct issues. The finalised questionnaire was translated into Arabic using classical back-translation methodology often employed in transcultural studies [14]. The participants who were included in the pilot study were then included in the whole study sample.

In order to assess the reliability and validity of the developed questionnaire, the newly-modified Arabic version was piloted on 30 randomly selected subjects. Before filling in the questionnaire, consent has been taken from the participants by getting their signature. Content and construct validity, as well as inter-rater reliability were high (kappa = 0.81).

To evaluate the statistical significance of differences among proportions of categorical data, Chi-square analyses were used. The non-parametric Fisher’s exact test (two-tailed) replaced the Chi-square test in cases of small sample size, where the expected frequency was less than 5 in any of the cells in 2 × 2 tables. The odds ratios (OR) and 95% confidence intervals (CI) obtained from logistic regression models were taken as the measures of predictors of non-willingness to perform pre-marital testing.

All statistical analyses were conducted using Statistical Package for Social Sciences (SPSS 19.0). The study was approved by the Regional Research and Studies Ethics Committee. The study was performed in accordance with the ethical standards laid down in the 1964 Declaration of Helsinki.

## Results

Table 1 shows the socio-demographic characteristics of the participants stratified by gender. Out of the total 400 participants, 233 (58.25%) were males. The majority were in their early adulthood, married, and reported to be employed with a relatively low income. The majority (64.5%) had acquired high school diploma. Compared to males, the number of females who were reported to be married and employed was significantly higher (P < 0.05).

**Table 1:** Socio-demographic characteristics of participants in the evaluation of knowledge and attitude towards pre-marital counseling by gender, Oman, 2010

**Characteristics**	**Total**	**Men**	**Women**	**P value**
	**(N = 400)**	**(N = 233)**	**(N = 167)**	
	**N (%)**	**N (%)**	**N (%)**	
**Age**				0.44
20 – 25	164 (41.0)	93 (39.9)	70 (42.0)	
26 – 30	153 (38.3)	86 (36.8)	67 (40.1)	
31 – 35	83 (20.7)	54 (23.2)	30 (17.9)	
**Marital status**				0.03
Married	237 (59.3)	124 (53.1)	114 (68.5)	
Single	154 (38.4)	106 (45.6)	46 (27.8)	
Divorced	7 (1.8)	2 (0.9)	5 (3.1)	
Widowed	2 (0.5)	1 (0.4)	1 (0.6)	
**Monthly income (OMR)**				0.11
<500	252 (63.0)	147 (63.1)	105 (62.9)	
500-1000	128 (32.0)	74 (31.8)	54 (32.3)	
>1000	12 (3.0)	10 (4.1)	2 (1.9)	
**Level of education**				0.19
High school level	258 (64.5)	161 (69.1)	97 (58.1)	
Graduate & above	142 (35.5)	72 (30.9)	70 (41.9)	
**Employment**				0.001
Unemployed	112 (28.0)	35 (15.1)	82 (49.1)	
Employed	283 (72.0)	198 (84.9)	85 (50.9)	

Table 2 shows indicators of knowledge of participants towards pre-marital carrier screening. Overall, the majority (89.3%) of the participants were aware of PMCS. Males appeared to be more aware of PMCS and its existence in their region. The main source of knowledge about PMCS was information from health workers, followed by periodical notes (monthly magazines), and then friends. Media and school were not endorsed to be an important source of knowledge. About one third of the participants reported that they were aware of the existence of all types of premarital medical tests. The majority reported that they were aware that hereditary disorders carry significant psychological burden on families. While awareness about SCD and G6PD was reasonably high, only about half of the participants reported awareness about thalassemia. Males had significantly increased awareness about SCD and G6PD than females, while females had increased awareness about thalassemia than males. The differences among genders in terms of awareness about these hereditary diseases were statistically significant.

**Table 2:** Indicators of knowledge about pre-marital by Gender, Oman, 2010

**Indicator**	**Total**	**Men**	**Women**	**P-value**
	**(N = 400)**	**(N = 233)**	**(N = 167)**	
	**N (%)**	**N (%)**	**N (%)**	
Heard of PMCS	357 (89.3)	201 (56.3)	156 (43.7)	0.01
Knew about regional PMCS clinic	271 (67.8)	148 (54.6)	123 (45.4)	0.51
Source of knowledge				0.003
Health workers	72 (18.0)	32 (44.4)	40 (55.6)	
Periodical notes	75 (18.8)	38 (50.7)	37 (49.3)	
Health educators	17 (4.3)	5 (29.4)	12 (70.6)	
Media	29 (7.3)	17 (58.6)	12 (41.4)	
School	13 (3.3)	10 (76.9)	3 (23.1)	
Friends	60 (15.0)	43 (71.7)	17 (28.3)	
New types of premarital tests	118 (29.5)	60 (50.8)	58 (49.2)	0.18
Aware of psychological burden of hereditary diseases on families	294 (83.8)	164 (55.8)	130 (44.2)	0.09
Heard of following diseases:				
Thalassemia	221 (55.3)	94 (42.5)	127 (57.5)	0.001
Sickle cell disease	335 (83.8)	186 (55.5)	149 (44.5)	0.01
G6PD	328 (82.0)	173 (52.7)	155 (47.3)	0.001
Knew about treatment for hereditary diseases	90 (22.5)	50 (55.6)	40 (44.4)	0.01
Knew that PMCS may reduce spread of heredity diseases	355 (88.8)	206 (58.0)	149 (42.0)	0.66

Table 3 shows indicators of attitude and practice among participants towards PMCS. Overall, only 10.5% of the participants, males in particular, preferred consanguineous marriages. The rate of consanguinity among married participants was 14.8%. More interestingly, 72.4% of the participants who are single did not prefer to get married from among their relatives whereas 27.6% preferred consanguineous unions. The majority preferred to avoid consanguineous unions so as to prevent the chance of developing hereditary diseases (79%) and the remainder to avoid family problems (8%).

**Table 3:** Indicators of attitude and practice towards pre-marital counseling by Gender, Oman, 2010

**Indicator**	**Total**	**Male**	**Female**	**P-value**
	**(N = 400)**	**(N = 233)**	**(N = 167)**	
	**N (%)**	**N (%)**	**N (%)**	
Preferred to marry a relative	42 (10.5)	34 (81.0)	8 (19.0)	0.02
Married people who did PMCS before marriage	34 (8.5)	18 (52.9)	16 (47.1)	0.81
Consanguinity among married couples	59 (14.8)	42 (71.2)	17 (28.8)	0.57
If future spouse is a carrier of a hereditary disease, still go for marriage	59 (14.8)	42 (71.2)	17 (28.8)	0.02
Willing to change decision about marriage based on PMCS results	250 (62.5)	135 (54.0)	115 (46.0)	0.03
Will advise future spouse to do PMCS	335 (83.8)	186 (55.5)	149 (44.5)	0.08
PMCS is important for self and spouse	338 (84.5)	194 (57.4)	144 (42.6)	0.31
Agreed to make PMCS compulsory	198 (49.5)	118 (59.6)	80 (40.4)	0.32
Unwilling to do PMCS test	122 (30.5)	78 (63.9)	44 (36.1)	0.14

The majority of the participants (84.5%) believed that PMCS is necessary and about half of them supported the view of making PMCS compulsory. The majority also reported that they would advise their spouse to take the premarital medical test. Also, more than 60% of the participants reported that they would consider results of PMCS prior to marrying a partner.

Nearly one third (30.5%) of the participants reported that they were not in favor of taking the test; whether married or single.

Lack of awareness, as the most common explanation, was reported among the married participants who did not perform the test. This constituted 36% of the participants. Others indicated that lack of testing centers (13%), no interest (10%), or lack of hereditary disease in the family (9%), not important (7%), or no relationship with partner (6%) as the reasons behind their failure to have sought PMCS.

Table 4 shows the results of the evaluation of associations between socio-demographic characteristics and unwillingness to perform pre-marital testing, as obtained from multivariate logistic regression modeling. The marital status was significantly associated with unwillingness to have pre-marital testing. The other socio-demographic factors were also predictors of unwillingness to have pre-marital testing, but the associations were not statistically significant. Overall, unwillingness to perform pre-marital testing tended to be associated with female gender, younger age, being single, less education, and increased income.

**Table 4:** Evaluation of predictors of non-willingness to perform pre-marital testing, Oman, 2010

**Characteristics**	**Refuse testing**	**Accept testing**	**OR**	**95% CI**	**P-value**
**Gender**					
Male	150 (37.5)	78 (19.5)	1.0	-	
Female	118 (29.5)	54 (13.5)	1.39	(0.89, 2.17)	0.14
**Age**					
20 – 25	115 (28.8)	50 (12.5)	1.0	-	
26 – 30	111 (27.8)	41 (10.3)	0.66	(0.38, 1.14)	0.14
31 – 35	50 (12.5)	33 (8.3)	0.56	(0.32, 0.99)	.04
**Marital Status**					
Single	149 (37.3)	87 (21.8)	1.0	-	
Got married	125 (31.3)	39 (9.8)	0.57	(0.37, 0.86)	.008
**Income**					
<500	172 (43.0)	82 (20.5)	1.0	-	
500-1000	90 (22.5)	44 (11.0)	1.95	(0.41, 9.42)	0.41
>1000	10 (2.5)	2 (0.5)	1.48	(0.27, 8.20)	0.65
**Level of education**					
School education	167 (41.8)	92 (23.0)	1.0	-	
Graduate & above	108 (27.0)	33 (8.3)	0.31	(0.02, 5.02)	0.41
**Employment**					
Unemployed	78 (19.5)	33 (8.3)	1.0	-	
Employed	192 (48.0)	97 (24.3)	1.02	(0.91, 1.15)	0.75

## Discussion

The application of PMCS is likely to bear enormous benefit in coming to grip the burden of congenital and inheritable genetic diseases. One of the common socio-cultural practices in some Arab/Islam countries is the high rate of consanguineous unions which have been reported to exceed 50% [15]. Due to the importance of PMCS in preventing hereditary diseases, this is the first community study designed to elicit Omanis’ knowledge, attitude, and perception towards PMCS.

In our study, the knowledge towards PMCS was adequate which had perhaps reflected high literacy among the majority of the participants. This finding is similar to a study done in Saudi Arabia which showed that 94.3% of the population studied expressed acceptance of PMCS as an effective tool to prevent genetic diseases [16]. A similar view has emerged from another non-western population. Oluwole and his colleagues in South-West Nigeria [17] have reported that 90.5% of the respondents were aware of PMCS.

Although most of the participants in this study heard about PMCS, half of them were unaware of premarital testing. The participants, mostly males, were not aware of thalassemia. In contrast, a study done in Saudi Arabia by Al Sulaiman et al. [18] was observed that the participants had a good knowledge about the nature of the tests. It was stated that 91% of the respondents in that study knew that blood testing is done for couples in PMCS and 94% of them knew that genetic disorders were the target for conducting the test [18].

The result of the pilot study conducted in South Batinah Governorate corresponds with the result in this study. After checking from the court registrations the number of subjects who did the premarital test before marriage, it has been found that a few individuals (2.9% in 2010) did the test before marriage in South Batinah which is similar to the findings among married couples (15% in previous years). On the other hand, the singles showed their willingness to take the test but they withdrew due to customs and traditions, as reported in our study. In addition, most of them showed resistance to consanguineous unions, but studies have shown that about half of Omani marriages are among relatives [7].

While Islam has sometimes been associated with the teachings of predestination, emerging view is that PMCS has been either condemned or condoned in Arab/Islamic societies. There appears to have lack of knowledge towards PMCS [19]. In our study, most of the participants believed that PMCS is important (86.5%) and 90.3% endorsed the view that PMCS has the potential to limit the spread of hereditary diseases. A similar finding obtained by Al-Aama [20] reported that about 96% of the respondents believed that PMCS is essential and 95% of them believed that PMCS would have limited the spread of hereditary disease.

The study found that males had statistically significant increased awareness of SCD and G6PD than females, while females had increased awareness about thalassemia than males. It is not clear why such difference would have occurred among gender groups. Nonetheless, this finding might have reflected the differential efforts in health education in the country. Also, it may be related to the way thalassemia and SCD are managed. Usually, management of thalassemia major would require frequent blood transfusion and iron chelating therapy which is usually applied by mothers at home [21]. On the contrary, the most common presentation of SCD is acute painful crises triggered by physical exercise [22]. This condition would require more active role from fathers in order to take the patient to emergency department to receive care or be admitted.

Controversy abounds on making PMCS mandatory for all individuals who would decide to get married. In our study, 41.5% of the participants disagreed while 50% agreed with the view of making PMCS mandatory. Al-Kahtani reported that 42% of the participants in the city of Riyadh endorsed the compulsory implementation of PMCS [15]. Al-Khaldi *et al.*[23] in Abha reported that 29% of their respondents supported the legalization of PMCS with equal numbers expressing dissenting. El–Hazmi *et al.*[5] surveyed seven cities in Saudi Arabia. The study reported that 86.9% of the respondents agreed that pre-marital examination should be made compulsory. Similar view has been noted echo in other studies in Saudi Arabia [20,18] and other non-western population such as those in Nigeria [17].

A mandatory strategy of PMCS has been employed in various Islamic countries including Bahrain [24], Saudi Arabia [16], Turkey and Iran where indications exist that the suggested spread of such handicapping conditions has been reduced. In Bahrain, for instance, PMCS is furnished for all prospective couples [24]. As the result of such campaign, the incidence of sickle cell anemia has gradually decreased in the country over time [25]. A similar observation has been noted in other countries [26,27]. Al-Gazali assessed the level of understanding the genetic advice at clinics and the attitude towards consanguineous marriages and preconception diagnosis among 100 couples in the UAE [28]. It was found that 75% agreed with carrier screening and preconception diagnosis, while only 10% agreed with prenatal diagnosis and abortion. It was concluded that there was a need to educate the general population to have an effective genetic counseling and preconception diagnosis. Al-Khaldi and colleagues explored the attitude of the students in a Health Sciences College towards premarital carrier screening in Saudi Arabia [23]. It was found that 70% of the students accepted premarital carrier screening. Legalization of premarital carrier screening was supported by only 19%. Students who refused justified their opinion by the misunderstanding of Islamic rules. The study concluded that there was a need for intensification of health education in which religious leaders should be involved in clarifying and correcting the misconception.

Many individuals choose to proceed with the marriage regardless of the results of the premarital testing [29]. In our study, it has been found that 15.3% (61 participants) decided to get married even in case of incompatible results. In addition, 22.3% (n = 89) of them refused to change their decision despite both couples being carriers of the same disorder. This finding might reflect the need to improve the impact of work policies at primary health centers. Currently, health education efforts at the primary health centers are carried out by medical and nursing staff while providing service for patients. There are no written policies or protocols that are standardized in practice which would identify the role of medical team members and expected outcomes of the process. Therefore, the outcome is dependent on personal commitment of staff and their availability. It might be worthwhile to develop a standard protocol related to pre-marital screening applied by healthcare staff.

The finding that significant proportion of participants refused to change their decision despite both couples being carriers of the same disorder would call to conduct a qualitative research to find out the reasons for these decisions. One very significant reason reported by Al-Aama in her study was the timing of the test [20]. The PMCS is usually done in the period between the engagement and marriage. An unexpected result may be ignored by couples or their families for various cultural, social, and emotional reasons [20]. Thus, the test should be done at an early stage so that both couples who decide to marry know the results prior to engagement. Another suggestion could be performing the test during the school or university stage, generally before 18 years, thus, making it mandatory for those who haven’t done it at these stages.

This study is not without limitations. First, the data was collected by self-administered method which resulted in a low response rate for some questions. Secondly, non-probability sampling method (convenience sampling) was used to collect the data and hence cannot be generalized as the result for the whole Governorate. Third, open questions have low response rate due to the greater effort involved in answering such questions. The relatively small sample size and low response rate might also have affected the power of the study to detect significant differences. As a matter of fact, not all observations were statistically significant across categories in the data analysis. Finally, the study was cross-sectional so we could not investigate cause and effect.

## Conclusions

In summary, this study represented that most of the participants who visited primary health care centres in the South Batinah Governorate have heard about PMCS while few of them have not. Test administration to married couples was low. These results could be changed through raising awareness among students by including PMCS in the secondary school curriculum. Health education should also be administered among adolescents to change their attitudes and opinions particularly before engagement. It might be worthwhile to develop standardized protocols that address knowledge and awareness about PMCS in daily clinical practice. In addition, increasing the number of educational programs in media like TV, radio and newspapers is an option which should be considered for mass outreach. An extensive study in all governorates in Oman should be done to get a clear understanding towards such an important health issue.

## Competing interests

The authors declare that they have no competing interests.

## Authors’ contributions

OAF formulated the study concept and collected data. He contributed to data analysis, literature review, and write-up of the manuscript. YMF and IG conceptualized the methods and contributed in reviewing results and write-up of the manuscript. YMF and OAF conceptualized the regression modelling techniques, reviewed the results and contributed to the write-up. IG and KAF contributed to the design and data collection in the field and contributed to the write-up. AO and SA revised the scientific background of the study and contributed to the literature review and write-up of manuscript, especially the Discussion. All authors read and approved the final manuscript.

## Pre-publication history

The pre-publication history for this paper can be accessed here:

http://www.biomedcentral.com/1471-2458/14/380/prepub
